# The Occurrence of Supernumerary Umbilical Cord Vessels: A Review for Practicing Clinicians

**DOI:** 10.3390/children12040418

**Published:** 2025-03-26

**Authors:** Éva Horváth-Varga, Eszter Hódi, Gyula Pásztor, Márta Katona, Hajnalka Orvos, Zita Gyurkovits

**Affiliations:** 1 Department of Obstetrics and Gynecology, Albert Szent-Györgyi Health Center, University of Szeged, 6725 Szeged, Hungary; hodi.eszter@med.u-szeged.hu (E.H.); kokutine.orvos.hajnalka@med.u-szeged.hu (H.O.); gyurkovits.zita@med.u-szeged.hu (Z.G.); 2 Department of Radiology, Albert Szent-Györgyi Health Center, University of Szeged, 6725 Szeged, Hungary; pasztor.gyula@med.u-szeged.hu; 3 Department of Pediatrics and Pedriatic Health Center, Albert Szent-Györgyi Health Center, University of Szeged, 6720 Szeged, Hungary; katona.marta@med.u-szeged.hu

**Keywords:** four-vessel umbilical cord, developmental abnormality, congenital heart disease

## Abstract

**Background**: The umbilical cord normally contains two arteries and one vein. The presence of supernumerary—four or five—umbilical cord vessels is a rare phenomenon, with few cases reported in the literature. The majority of cases are detected postnatally. However, given their potential association with developmental abnormalities, primarily severe cardiac anomalies and genetic disorders, the prenatal diagnosis of supernumerary umbilical cord vessels may have clinical relevance. **Methods**: A review of the clinical phenomenon of the four-vessel umbilical cord and its complications was conducted using case studies and literature reviews in PubMed from 1977 to the present and in Google Scholar from 1966 to the present. **Results**: Among the 24 reported cases, 7 cases were associated with malformations, 8 cases were detected antenatally, and 16 cases postpartum. Among the eight antenatally diagnosed cases, only one had a congenital malformation, hydrops fetalis. Among the postnatally diagnosed cases, six had congenital abnormalities: three were cardiovascular, two were associated with hydrops, urinary, gastrointestinal, and skeletal disorders, hypoplastic corpus callosum, and dysmorphic facial features. **Conclusions:** Four-vessel umbilical cords are more frequent than previously thought, as they can be easily overlooked during the mandatory ultrasound examination. A review of the literature revealed a correlation between supernumerary umbilical cord vessels and major congenital malformations, underscoring the significance of prenatal diagnosis; however, the four-vessel cord may not always be indicative of a serious condition.

## 1. Introduction

Umbilical cord inspection is part of the initial postnatal medical examination of a newborn. Several conditions may indicate low fetal oxygenation, including the twisting of the umbilical cord around the neck, limbs, or trunk; the presence of a true knot; or the meconium staining of the cord. Additionally, an abnormal number of blood vessels in the cord may suggest genetic and developmental abnormalities.

The umbilical cord normally contains two arteries and one vein, and its intrauterine examination is part of mandatory screening. While single umbilical arteries (SUAs) are relatively common, with an incidence of 0.5–6.0%, the presence of four (0.11–0.5%) or five blood vessels in the umbilical cord is considerably less frequent [1,2,3,4]. Supernumerary vessels may present as an artery, a vein, or an omphalomesenteric duct. Among cases of four-vessel umbilical cords (FVUCs), the persistent right umbilical vein (PRUV) represents the most prevalent variant, where the extra lumen is a vein. Less commonly, the cord consists of three arteries and one vein. The clinical significance of FVUCs warrants careful consideration, as it has been associated with severe developmental abnormalities in multiple case reports and systematic reviews [5,6].

## 2. Materials and Methods

A review of the clinical phenomenon of the four-vessel umbilical cord and its complications was conducted using case studies and literature reviews. A search was conducted for the phrase ‘four-vessel umbilical cord’ in the literature available in PubMed from 1977 to the present and in Google Scholar from 1966 to the present. Studies written in English that reported the four-vessel umbilical cord were included.

## 3. Results and Discussion

During embryogenesis, the right umbilical vein occludes around the sixth gestational week [5], while the left umbilical vein and the two arteries form the umbilical cord vessels. Occasionally, the obliteration of the right vein does not occur, resulting in the persistence of both the left and right umbilical veins. The etiology of a PRUV can be multifactorial, involving genetic, environmental, and nutritional elements. Commonly, nutritional deficiencies such as folic acid play a crucial role, particularly during the early stages of embryonic development. Teratogenic effects, such as exposure to retinoic acid or other harmful substances during the first trimester, are significant as well [1]. The reported incidence of PRUV varies from 0.11 to 0.5% due to differences in the experience of ultrasonographers, as the abnormality can be easily overlooked [2,3,4]. Improvements in prenatal ultrasound techniques have increasingly enabled the diagnosis of umbilical cord abnormalities. The diagnosis of such conditions may provide clues on general fetal well-being and also predict potential complications.

In 2012, Koolhaas et al. reported the following incidences of PRUV-associated abnormalities: cardiovascular (60.3%), gastrointestinal (12.8%), musculoskeletal (7.7%), renal (15.4%), and cerebral (15.4%) defects [7]. Less frequent associations included hypospadias, retrognathia, VACTERL syndrome, goiter, poly/asplenia, trisomy 18, and Turner syndrome [7].

PRUVs present in two distinct forms: intrahepatic and extrahepatic types. The intrahepatic form is more common (75–95%), characterized by the right umbilical vein flowing into the portal vein and forming the ductus venosus [6]. Its prevalence was reported to be 0.13% by Lide et al. in 2016, and 0.38% by Toscano et al. in 2019 [6,8]. The intrahepatic type can present as an isolated finding (76.7%) or in association with other anomalies (23.3%), including cardiovascular, genitourinary, or central nervous system abnormalities [6,8].

The extrahepatic type, characterized by an absent ductus venosus, is less common [9]. With this variant, the right umbilical vein completely bypasses the liver and drains directly into the right atrium, the intracardiac portion of the inferior vena cava, or the iliac veins. This variant is associated with fetal congestive heart failure due to cardiac overload and hemodynamic stresses, as well as several diverse major congenital anomalies [9]. With the extrahepatic type, the ductus venosus is lacking, resulting in anomalous venous drainage from the placenta to one of three different places. Fetal hydrops may be caused by all the blood in the umbilical cord bypassing the liver and flowing directly to the heart. Two main types of an absent ductus venosus can be distinguished: (i) the umbilical vein bypasses the liver completely, causing overflow into the heart and the underperfusion of the liver, and (ii) the umbilical vein drains into the portal vein, causing all the umbilical blood to pass through the hepatic sinusoids, thus the blood from the umbilical vein overflows the hepatic sinusoids, resulting in the overperfusion of the liver. The former pattern is interpreted as a fundamental abnormality of the venous system, in which the umbilical veins are not integrated into the vitelline venous system [10,11]. Hepatic hyperperfusion and portal hypertension can cause damage to liver cells and impaired fetal plasma protein synthesis and secretion. This may contribute to fetal hypoproteinemia and may be a cause of hydrops [12,13]. The developing liver may have adaptive potential to compensate for hemodynamic changes due to the absence of the ductus venosus.

The absence of a ductus venosus can cause problems in the hemodynamics of the fetal circulatory system, and often presents as hydrops fetalis, generalized skin edema, ascites, pleural effusion, or heart failure. This could lead to detrimental consequences for the fetus such as premature delivery or increased risk for perinatal mortality. However, in 2013, Firdouse et al. reported two cases without any malformation, despite the absence of the ductus venosus [14]. In summary, the description of the anatomy of the fetal venosus system and the developmental anomalies leading to FVUCs is very complex [15].

In Table 1 and Table 2, we present cases of a four-vessel umbilical cord with and without associated malformations. The literature review suggests that supernumerary umbilical cord vessels, notably four-vessel umbilical cords, are associated with major congenital malformations, which is why prenatal diagnosis is very important.

The ultrasound scan allows for a comprehensive examination of the fetal anatomy in cases where there is an abnormal number of vessels in the umbilical cord [26]. An umbilical cord containing four vessels does not always indicate an unfavorable perinatal outcome. It is generally observed that cases with an umbilical cord containing three arteries and one vein tend to have a more favorable outcome [36]. Conversely, Schimmel et al. emphasize the necessity for thorough investigations following the birth of an infant with a four-vessel cord, aiming to detect associated abnormalities [24]. However, they also note that this finding does not necessarily imply a negative outcome [24]. These findings suggest that the prenatal screening of umbilical cord blood vessels may be recommended for the detection of fetal abnormalities [23]. Hoppen et al. recommend that in all cases of hydrops fetalis, the venous system should be evaluated prenatally and/or immediately postnatally using ultrasonography to detect any abnormality in venous circulation, especially the absence of the ductus venosus [17]. In their study in 2011, Avent et al. examined the trajectory of the two umbilical veins from the fetal abdominal cavity to the portal system, observing the presence of two umbilical veins [27]. They found that the right umbilical vein contributed to part of the right portal system, while the left umbilical vein contributed to parts of both the left and right portal systems. The authors hypothesized that the connection between the right anterior portal vein and the right posterior portal vein generally occurs after the right umbilical vein has regressed, suggesting that this may explain the observed heterogeneity of the connection between the right anterior and right posterior portal veins. In the absence of further anomalies, the prognosis appears favorable. As Lei et al. demonstrated, the presence of supernumerary veins and varices can serve as a predictor of adverse prenatal outcomes, particularly in cases involving other congenital anomalies [30]. In their study, the authors emphasize the importance of the meticulous evaluation of the sagittal/cross-section of the umbilical cord, with a focus on the number of umbilical veins present at both the free loop and the umbilical ring. Furthermore, they underscore the necessity for detailed examination of the intra-abdominal umbilical vein during screening procedures. In the presence of FVUCs, comprehensive antenatal care and serial follow-up examinations are necessary to exclude other congenital anomalies, hydrops, and varix thrombosis. Kurakazu et al. emphasize the necessity of evaluating the number of umbilical cord blood vessels during the second trimester using ultrasound with color Doppler at a minimum of three sites: the fetal abdomen and the insertion sites of the placenta, as well as the free loop of the umbilical cord [31]. They argue that the prenatal diagnosis of isolated, supernumerary umbilical cord vessels frequently fails. However, the presence of supernumerary cord blood vessels has been associated with fetal congenital anomalies. Consequently, it is recommended that the number of blood vessels in the umbilical cord is investigated, as the detection of such abnormalities may facilitate a prenatal diagnosis of other congenital anomalies.

Among the 24 reported cases, 7 cases were associated with malformations, sometimes with multiple. Eight cases were detected antenatally between the 22nd and 36th gestational week and sixteen cases postpartum. Among the antenatally diagnosed eight cases, only one had a congenital malformation, hydrops fetalis. Among the postnatally diagnosed cases, six had congenital abnormalities: three were cardiovascular, two were associated with hydrops, urinary, gastrointestinal, and skeletal disorders, hypoplastic corpus callosum, and dysmorphic facial features. Only one case required the termination of the pregnancy; in this case, the associated abnormality was hydrops. The Apgar scores and FVUCs showed no clear association, but newborns with hydrops had low Apgar scores in both cases. There was one case of genetic abnormality: the trisomy of chromosome 18 was confirmed. The presence of risk factors during pregnancy occurred in seven cases, and intrauterine growth restriction in eight cases. The mode of delivery was cesarean section in all but two cases of neonates with developmental anomalies.

We also present the case of a 35-year-old woman at 39 weeks of gestation (GW) who gave birth to a newborn via cesarean section due to maternal indication at the University of Szeged, Hungary. The neonate weighed 4250 g, their Apgar scores were 9, 10, and 10 at 1, 5, and 10 min, respectively. Their arterial umbilical cord blood pH was 7.30, and lactate was at 2.4 mmol/L. The maternal history included two spontaneous abortions, and the paternal line had trisomy 21.

At the first-trimester screening (at 12 GW), intrauterine ultrasound examination revealed a 5.8 × 2.9 × 4.6 mm large septal, fluid-filled, jugular cyst on the neck with a normal nuchal translucency (NT), 2.2 mm. The follow-up examination at 19 GW showed the complete regression of the cystic hygroma and no other anomalies. As cystic hygroma is usually associated with an increased risk of fetal chromosomal and developmental abnormalities, in our case, genetic counseling and testing, including non-invasive prenatal tests, chorionic villus sampling, and amniocentesis were carried out, and showed no major chromosomal abnormalities. Quantitative molecular genetic analysis was also negative. Chromosome analysis confirmed a normal 46, XX karyotype. The fetal echocardiologic ultrasound showed no alterations.

**Figure 1:**
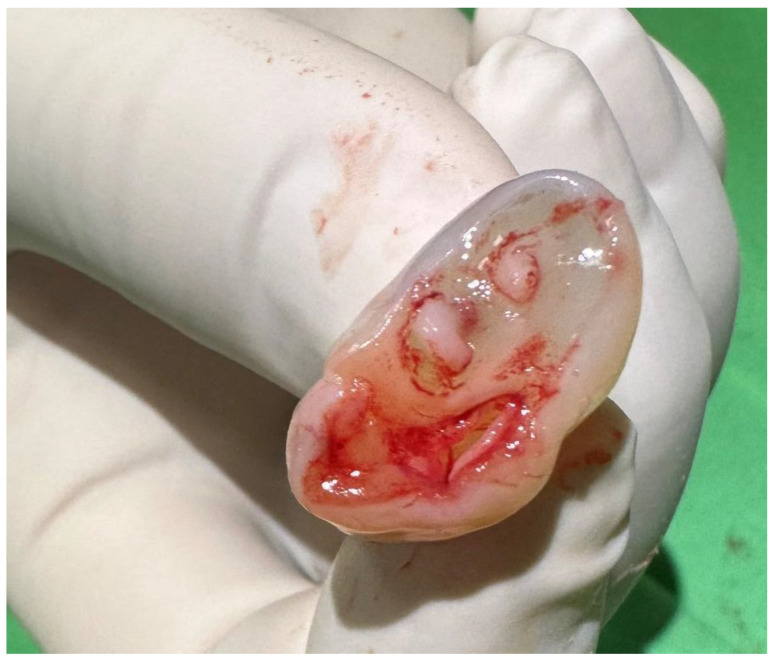
Umbilical cord with two arteries and two veins.

After birth, a cross-section of the umbilical cord revealed four vessels, two arteries, and two veins (Figure 1). In the first examination of the newborn, a systolic murmur of grade II/VI in the left lower sternal border was detected, otherwise, the physical examination was normal. The ultrasound of the neck, chest, and abdomen was also negative. No structural abnormality was seen during echocardiographic examination. The neonate was discharged on the third day of life without any complications.

## 4. Future Directions

In the current era, with a wide spectrum of non-invasive examination methods, the initial postnatal medical examination of the newborn remains a fundamental and straightforward procedure. The presence of a four-vessel umbilical cord can be detected during pregnancy; however, when three vessels are observed, the examiner often ceases further investigation, given the rarity of supernumerary umbilical cord vessels.

## 5. Conclusions

In summary, FVUCs are more frequent than previously thought, as they can be easily overlooked during the mandatory ultrasound examination. If supernumerary umbilical cord vessels are detected, prenatal diagnosis, genetic testing, and fetal echocardiography should be carried out. Both intra- and extrauterine screening of the number of umbilical cord blood vessels is of paramount importance in conducting the necessary fetal and newborn examinations to rule out any accompanying genetic or developmental abnormalities. A review of the literature revealed a correlation between supernumerary umbilical cord vessels and major congenital malformations, underscoring the significance of prenatal diagnosis; however, the four-vessel cord may not always be indicative of a serious condition.

## Figures and Tables

**Table 1:** Reported patients with four-vessel umbilical cords and associated malformations.

Article	Age of Mother	Risk Factors	GW at Birth (Week)	Mode of Delivery	Gender	Weight (g)	Apgar Score (1 and 5 min)	Diagnosis of FVUC	Type of FVUC	Associated Mal-Formations	Intrauterine Growth Restriction	Fetal Karyotype
Painter (1977) [16]	32	NR	37	vag	M	1550	1–7	pp	2a, 2v	complete thoracic ectopia cordis	NR	IUD
										asymmetrical bifid liver		
										severe bilateral cleft lip and palate with absent soft palate and uvula		
										cecum mobile		
Hoppen (2000) [17]	32	polyhydramnion	34	cs	M	1940	1–7–8	34 GW	2a, 2v	hydrops	no	normal
		proteinuria										
		agenesis of ductus venosus										
		2 v.umb. drained into the right atria										
Karatza (2011) [18]	16	NR	40	cs	F	NR	1–2	pp	2a, 2v	hydrops	no	normal
										hypertrophic cardiomyopathy		
Puvabanditsin (2011) [5]	20	NR	39	cs	F	2730	8–9	pp	2a, 2v	heterotaxy syndrome	yes	normal
Degirmencioglu (2012) [19]	22	NR	32	cs	F	1240	4–6	pp	2a, 2v	esophagus atresia	yes	trisomy 18
										hypoplastic corpus callosum		
										dysmorphic facial features		
										clenched hands		
										rocker bottom feet		
										intracranial hemorrhage		
Premkumar (2023) [20]	26	none	37	vag	M	2540	7–8	pp	3a, 1v	d-TGA	NR	
										single coronary artery		
										left-side urinary tract dilatation		

cs: cesarean section, d-TGA: dextro-transposition of the great arteries, F: female, FVUC: four-vessel umbilical cord, GW: gestation week, IUD: intra uterine death, M: male, NR: not reported, pp: postpartum, and vag: vaginal.

**Table 2:** Reported patients with four-vessel umbilical cords without associated malformations.

Article	Age of Mother	Risk Factors	GW at Birth (Week)	Mode of Delivery	Gender	Weight (g)	Apgar Score (1 and 5 min)	Time of Diagnosis of FVUC	Type of FVUC	Intrauterine Growth Restriction	Fetal Karyotype
Murdoch (1966) [21]	16	NR	39	forceps	M	2806	10-10	pp	2a, 2v	NR	NR
Rodrigez (1984) [22]	30	di-zygote twin	NR	cs	F	3090	1–7	pp	2a, 2v	no	NR
Aoki (1997) [23]	NR	NR	37	vag	F	3016	9, NR	29 GW	2a, 2v	no	NR
Aioki (1997) [23]	NR	NR	39	vag	F	3588	9, NR	23 GW	2a, 2v	no	NR
Schimmel (1998) [24]	NR	IVF	30	NR	NR	1320	1–7	pp	2a, 2v	yes	NR
Paize (2006) [25]	NR	NR	27	vag	M	NR	NR	pp	2a, 2v	no	NR
Perez-Cosio (2008) [26]	37	SLE	38	cs	M	3240	8–9	32 GW	2a, 2v	no	NR
Avent (2011) [27]	33	NR	39	cs	F	3780	9–10	22 GW	2a, 2v	no	normal
Koolhaas (2012) [7]	26	meconium-stained amniotic fluid	41	vag	M	4420	8–9	pp	2a, 2v	yes	NR
Panda (2013) [28]	37	oligohydramnion	33	vag	F	1400	NR	pp	1a, 2v	yes	IUD
Hoh (2015) [29]	NR	no	38	NR	M	NR	NR	22 GW	3a, 1v	NR	NR
Lei (2017) [30]	31	NR	38	forceps	M	2660	10–10	36 GW	2a, 2v	yes	normal
Kurakazu (2019) [31]	37	NR	38	NR	F	2726	NR	36 GW	2a, 2v	no	NR
Kaur (2020) [32]	24	none	34	vag	NR	2000	NR	pp	2a, 2v	NR	NR
Damiani (2021) [33]	NR	IUGR, bicornuate uterus	37	NR	NR	2500	NR	pp	4a, 1v	yes	NR
Arora (2022) [34]	NR	none	38	vag	NR	NR	5–6–8	pp	2a, 2v	NR	NR
Allen (2024) [35]	22	none	37	vag	NR	2360	9–10	pp	2a, 2v	NR	NR
Our case	35	none	39	cs	F	4250	9–10–10	pp	2a, 2v	no	NR

cs: cesarean section, F: female, FVUC: four-vessel umbilical cord, GW: gestation week, IUD: intra uterine death, IUGR: intrauterine growth restriction, M: male, NR: not reported, pp: postpartum, SLE: Systemic lupus erythematosus and vag: vaginal.

## Data Availability

The original contributions presented in this study are included in the article. Further inquiries can be directed to the corresponding author.
